# An Evaluation of the BEEHAVE Model Using Honey Bee Field Study Data: Insights and Recommendations

**DOI:** 10.1002/etc.4547

**Published:** 2019-09-24

**Authors:** Annika Agatz, Roland Kuhl, Mark Miles, Thorsten Schad, Thomas G. Preuss

**Affiliations:** ^1^ ibacon, Rossdorf Germany; ^2^ Crop Science Division, Bayer Monheim Germany

**Keywords:** Environmental modeling, Hazard/risk assessment, Landscape ecology, Population modeling, stressors, BEEHAVE, BEESCOUT, *Apis mellifera*

## Abstract

A lack of standard and internationally agreed procedures for higher‐tier risk assessment of plant protection products for bees makes coherent availability of data, their interpretation, and their use for risk assessment challenging. Focus has been given to the development of modeling approaches, which in the future could fill this gap. The BEEHAVE model, and its submodels, is the first model framework attempting to link 2 processes vital for the assessment of bee colonies: the within‐hive dynamics for honey bee colonies and bee foraging in heterogeneous and dynamic landscapes. We use empirical data from a honey bee field study to conduct a model evaluation using the control data set. Simultaneously, we are testing several model setups for the interlinkage between the within‐hive dynamics and the landscape foraging module. Overall, predictions of beehive dynamics fit observations made in the field. This result underpins the European Food Safety Authority's evaluation of the BEEHAVE model that the most important in‐hive dynamics are represented and correctly implemented. We show that starting conditions of a colony drive the simulated colony dynamics almost entirely within the first few weeks, whereas the impact is increasingly substituted by the impact of foraging activity. Common among field studies is that data availability for hive observations and landscape characterizations is focused on the proportionally short exposure phase (i.e., the phase where colony starting conditions drive the colony dynamics) in comparison to the postexposure phase that lasts several months. It is vital to redistribute experimental efforts toward more equal data aquisition throughout the experiment to assess the suitability of using BEEHAVE for the prediction of bee colony overwintering survival. *Environ Toxicol Chem* 2019;38:2535–2545. © 2019 The Authors. *Environmental Toxicology and Chemistry* published by Wiley Periodicals, Inc. on behalf of SETAC

## Graphical abstract

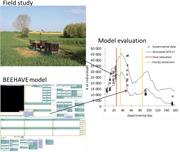

## INTRODUCTION

In recent years the European Union has increased the demand for ecotoxicological testing with honey bees for the environmental risk assessment conducted in preparation of the registration of pesticides on the market. Besides the acute oral toxicity test (Organisation for Economic Co‐operation and Development 1998a) and the acute contact toxicity test (Organisation for Economic Co‐operation and Development 1998b) for adult bees formalized in 1998, additional test methods have been established to cover chronic exposure of adult bees (Organisation for Economic Co‐operation and Development 2017) and exposure of bee larva (Organisation for Economic Co‐operation and Development 2013). Beyond these laboratory tests, semifield (Organisation for Economic Co‐operation and Development 2014) and field (European Food Safety Authority 2013) tests are conducted with whole bee colonies to assess pesticide impacts in a more environmentally realistic setting. Whereas the former represents a worst‐case exposure without alternative foraging sources for bee colonies during the exposure phase, the latter is often associated with challenges in the analysis and interpretation of gathered data. Analysis of data is challenging because of the complexity of factors influencing colony dynamics (e.g., diseases, parasites, weather, landscape, and beekeeping practices) that can act either individually or in combination. Further complexity is given by the inability to conduct field experiments with a control and treatment trial at exactly the same location because of potential cross‐contamination. The complexity makes the interpretation of field studies, and with that the risk assessment for bees, difficult. Additional contributions to the difficulty of risk assessment for bees are the enormous efforts, challenges, and costs associated with field studies. The inability to conduct such studies for all pesticides, their different usages, and at different climatic zones adds to the challenges. For support, focus has been given to the development and testing of modeling approaches that can be used for the risk assessment of plant protection products on bee colonies. Though earlier models for the simulation of colony performance and colony dynamics exist (BEEPOP, DeGrandi‐Hoffman et al. 1989; HoPoMo, Schmickl and Crailsheim 2007), BEEHAVE (Becher et al. 2014) is the first model attempting to link within‐hive dynamics and foraging in heterogeneous and dynamic landscapes, which is vital for environmental realism in the prediction of colony dynamics.

The European Food Safety Authority (EFSA) has conducted a stepwise evaluation of the BEEHAVE simulation model for risk assessment of multiple stressors at the landscape level, using its opinion on good modeling practice (European Food Safety Authority 2014), with a view to assessing its suitability for use in a regulatory context (European Food Safety Authority 2015). It was concluded that BEEHAVE performs well in terms of simulating bee colony dynamics. Furthermore, it was mentioned that the model needs an extension with an ecotoxicological submodule. This would allow the assessment of impacts from plant protection products on bee colonies, including the interaction of such impacts with other stressors like the varroa mite and viruses. Another suggestion was the consideration of a more realistic representation of the landscape within the model and a wider representation of different environmental scenarios to represent Europe with its different geopolitical zones.

There were recent efforts to address the mentioned shortcomings of the BEEHAVE model described in the EFSA scientific opinion to increase its usability in a regulatory context and for risk assessment of multiple stressors at the landscape level. A submodel (BEESCOUT), allowing the creation of a landscape and its parameterization ready to be used within the BEEHAVE model, was published by the model developers (Becher et al. 2016). This module also allows investigation of the influence of different scouting behaviors of bees on the forager activity and the resulting colony dynamics. Furthermore, work was conducted to use BEEHAVE in the context of investigating the impact of plant protection products on bee colony dynamics. Such work included exploration of the pesticide protection goal for bee worker losses at different forage qualities (Thorbek et al. 2017a), to assess the impact of pesticide‐induced sub‐lethal effects on bee workers (Thorbek et al. 2017b) and to assess the impact of pesticide residues brought back to the hive via pollen of seed‐treated corn when simulating real agricultural landscapes in the US Midwest (Schmolke et al. 2019). To our knowledge, a comparison of BEEHAVE simulations with empirical data from field studies has not been conducted. In the present study, we used control data of a field study conducted in 2014 in Germany (Heimbach et al. 2016; Rolke et al. 2016) to 1) identify whether the landscape characterization and parameterization with data from the field studies, the open literature, and the use of BEESCOUT (Becher et al. 2016) are suitable to simulate bee colony dynamics with BEEHAVE in agreement with field observations over one season; 2) investigate how different theoretical scouting behaviors of bees available in BEESCOUT affect forager activity and the resulting colony dynamics in BEEHAVE; and 3) present improvement options for field studies with bee colonies, to allow retrospective modeling with BEEHAVE and the development of Europe‐wide standard modeling scenarios in the future.

## MATERIALS AND METHODS

### Summary of the field study used

Full details on the field study used can be found in the literature (Heimbach et al. 2016; Rolke et al. 2016). In summary, the entire field study consisted of 96 beehives (derived from sister queens almost 1 yr prior the start of the actual experiment) that were split into 2 groups (control [C] and treatment [T]). Hives were placed at 6 locations (CA–CF and TA–TF) with 8 hives each (e.g., CA‐1–CA‐8) in either the control or the treatment site for the start of the experiment. Both sites were specified by an area with a 9‐km diameter in the vicinity of Sternberg, Mecklenburg‐West Pomerania (Mecklenburg‐Vorpommern), in northern Germany. The control and treatment sites differed in the fact that oilseed rape (OSR) within the sites was drilled with seeds that received no treatment in the former case and were treated with clothianidin in the latter case. Both areas had approximately the same oilseed rape coverage. The 6 hive locations within each site were specified by their difference in the immediate surrounding landscape. Some hives were placed close to an oilseed rape field and others farther away. Hives were brought to their locations on 22 April 2014, and the numbers of adult bees, number of open and capped worker broods, number of open and capped drone broods, and number of fallen varroa mites were determined on a weekly basis. Virus infection rates, honey production, pollen and honey composition, and the pesticide concentration in the field, pollen, and honey were measured less frequently. After 4 wk of colonies being able to forage and develop at their specific site within the landscape (from now on referred to as L1), hives from both the control and the treatment sites were relocated to a postexposure site in Erlensee (Hessen, Germany, from now on referred to as L2). There, hives were placed at one of 4 locations within a former airbase. At location L2, colonies were allowed to forage and develop for an additional 17 wk, and hive monitoring continued frequently. The field study included an ecological landscape characterization. Focus was given to plants flowering during the experimental phase and plants known to serve as food for honey bees. This characterization was conducted for the first phase of the experiment only (i.e., when hives where located in L1). We used all information from the 8 control hives (CA‐1–CA‐8) that were placed at one location within the first study period (L1, location CA) and afterward relocated to either of the 4 locations at the airbase in Erlensee (L2, 2 hives per relocation site [a, b, c, and d]).

### Overview of the BEEHAVE model

The honey bee colony model, BEEHAVE (Becher et al. 2014), was developed to explore how various stressors (varroa mites, virus infections, impaired foraging behavior, changes in landscape structure and dynamics, and pesticides), when acting individually or in combination, influence the performance of single managed colonies of honey bees. The model consists of 3 integrated modules (a colony module, a mite module, and a foraging module) and runs for 1 yr or multiple years in a daily time step (always starting on Julian day 1; Becher et al. 2014). Currently, the model does not allow for the exploration of multiple colonies in a landscape that interact with each other, and the impact of pesticides on the colony dynamics is not explicitly incorporated via a mechanistic effect modeling approach. The model is accompanied by a submodel (BEEHAVE‐Weather) that can be used to create weather files needed for BEEHAVE simulations. These weather files provide information on when and for how long the site‐specific weather conditions are suitable for foragers to be actively foraging in a specified landscape.

The colony module describes in‐hive processes that produce colony structure (number of individuals specified as eggs, larvae, pupae, and in‐hive bees of both workers and drones) and dynamics within the hive (egg‐laying, brood care).

The mite module processes the dynamics of a varroa mite population in the colony. This module is interlinked with the bee colony via varroa causing mortality to pupa and adult bees and serves as virus vector.

The foraging module is a cohort‐based model that simulates foraging of adult bees on user‐defined food sources. Properties of these food sources (i.e., flower patches within user‐defined distances to the hive) are their chance to be detected by a foraging bee and their nectar and pollen availability (i.e., mass over time). The foraging process during a day depends on the quality of food sources in the landscape, the weather, and several colony internal processes (e.g., the honey and pollen store). The foraging process is separated into one or several foraging trips and thus operates on a timescale of minutes. BEEHAVE itself incorporates some simple food source scenarios, and a few more complex but theoretical landscapes that can directly be used to run the model. An external model called BEESCOUT (Becher et al. 2016) can be used to create BEEHAVE readable input files for more complex landscapes that can be used as hive surroundings within the foraging module of BEEHAVE.

### Overview of the BEESCOUT model

BEESCOUT (Becher et al. 2016) is a model simulating bee scouting behavior according to different theoretical scouting strategies or combinations of these (e.g., colony, mixed, random) that can be used to characterize landscapes in terms of food availability (i.e., nectar and pollen availability across a landscape) and the probability of these food sources to be detected by scouting bees in proximity of a bee colony placed in that landscape. The landscape used in BEESCOUT is user‐defined and can be of variable complexity. Ranging from only one or 2 food patches that are located a specific distance from the hive to realistic landscapes with different landscape attributes (e.g., arable fields, field margins, kettles [depressions in the landscape formed by partially or fully buried ice masses that melted], forests, hedges, groves of trees, urban areas, surface waters) represented as color‐coded patches. The model allows loading landscape pictures (for example, from satellites) or creating landscapes manually to subsequently define the landscape scale and characterize the user‐defined landscape, accounting for proximity to the beehive, its food availability for bees, and the habitat‐specific detection probability for scouting bees. One limitation of the current version of BEESCOUT is that the landscape can contain an almost unlimited number of attributes (i.e., a large number of unconnected fields). But the number of habitat types within that landscape (e.g., oilseed rape, maize, forest) within one run is limited to surface water and 4 habitat types that can be parameterized with a type‐specific food availability. Food availability in BEESCOUT is defined by the start and end dates of food provision at this habitat type (i.e., start and end of the oilseed rape flowering period), the average nectar and pollen availability, the sugar content of nectar, and the average handling time of a forager bee to successfully collect a full nectar or pollen load. The current way of implementing the food availability in a habitat and the way this information is processed do not allow the implementation of more complex food availability in a habitat without substantial alteration in the BEESCOUT model code. A more detailed description of the BEESCOUT model can be found in the literature (Becher et al. 2016). BEHAVE and the submodels were implemented in the freely available and easy to learn software platform NetLogo (Wilensky 1999).

### BEEHAVE alterations made

No substantial changes to the code of BEEHAVE that would alter the behavior of honey bees or varroa mites were made. Thus, the actual processes and overall model behavior remained unchanged. All additions made to the original BEEHAVE code are provided in the Supplemental Data. We added switches and input options at the interface and a diagram option to allow 1) starting a model run at any specific day of the year, 2) specifying the starting conditions of the colony at the start of the experiment, and 3) more temporal flexibility in beekeeper activities (i.e., timing of honey extraction and hive feeding). For details on the switches and inputs added, see Supplemental Data, Table S1.

Furthermore, we added 2 new procedures into the model that set the initial conditions of the colony and create the different cohorts of bees with specific ages at the beginning of the simulation. For the latter, we used an assumption regarding the age distribution within each cohort because such information was not available from data gathered in the field study. We assumed that within each brood cohort (i.e., eggs, larvae, and pupae of workers and drones) and independent of the total number of individuals within that cohort each possible age class had the same relative number of individuals at the beginning of the simulation. For adults the number of bees was used to create one cohort with an age of 5 d. This already resulted in availability of nursing and foraging bees and thus prevented the modeled colony from collapsing within the first few days of the simulation.

### Deriving colony starting conditions for modeling

All starting conditions used for the BEEHAVE model runs conducted are presented in Table 1. Further details on how these values were calculated can be found in the Supplemental Data.

**Table 1:** Colony‐specific starting conditions used for all BEEHAVE runs^a^

Hive ID	Adult bees	Worker pupa	Worker larvae	Worker eggs	Drone pupa	Drone larvae	Drone eggs	Pollen (kg)	Honey (kg)
CA‐1	13 000	13 900	1314	5986	0	702	3198	2.4	31.8
CA‐2	13 228	9200	1386	6314	0	655	2985	3.3	37.2
CA‐3	13 748	18 200	666	3034	0	655	2985	3.0	38.9
CA‐4	12 058	16 800	1926	8774	0	562	2558	3.7	43.2
CA‐5	13 098	19 300	972	4428	0	468	2132	2.6	32.0
CA‐6	14 983	22 900	342	1558	325	725	3305	2.3	30.8
CA‐7	14 788	18 300	612	2788	0	725	3305	2.3	31.0
CA‐8	14 853	15 100	504	2296	0	725	3305	2.5	30.2
Average	13 719	16 713	965	4397	41	652	2971	2.8	34.4

^a^ Starting conditions were applied on Julian day 116 (26 April).

To create weather files needed by BEEHAVE, we used the submodel BEEHAVE‐Weather (Becher et al. 2014) and daily data on sunlight hours and air temperature that originated either from the field study itself or from weather stations near the experimental site. For the first part of the experiment (hives were located at L1) the sunlight hours were taken from a weather station in Schwerin (Deutscher Wetterdienst, Station 4625), and temperature measurements were made at the actual hive location as part of the experiment. For the second experimental phase (hives were located at L2) maximum air temperature and sunlight hours originated from a weather station in Erlensee.

### Landscape characterization for BEESCOUT modeling

**Figure 1:**
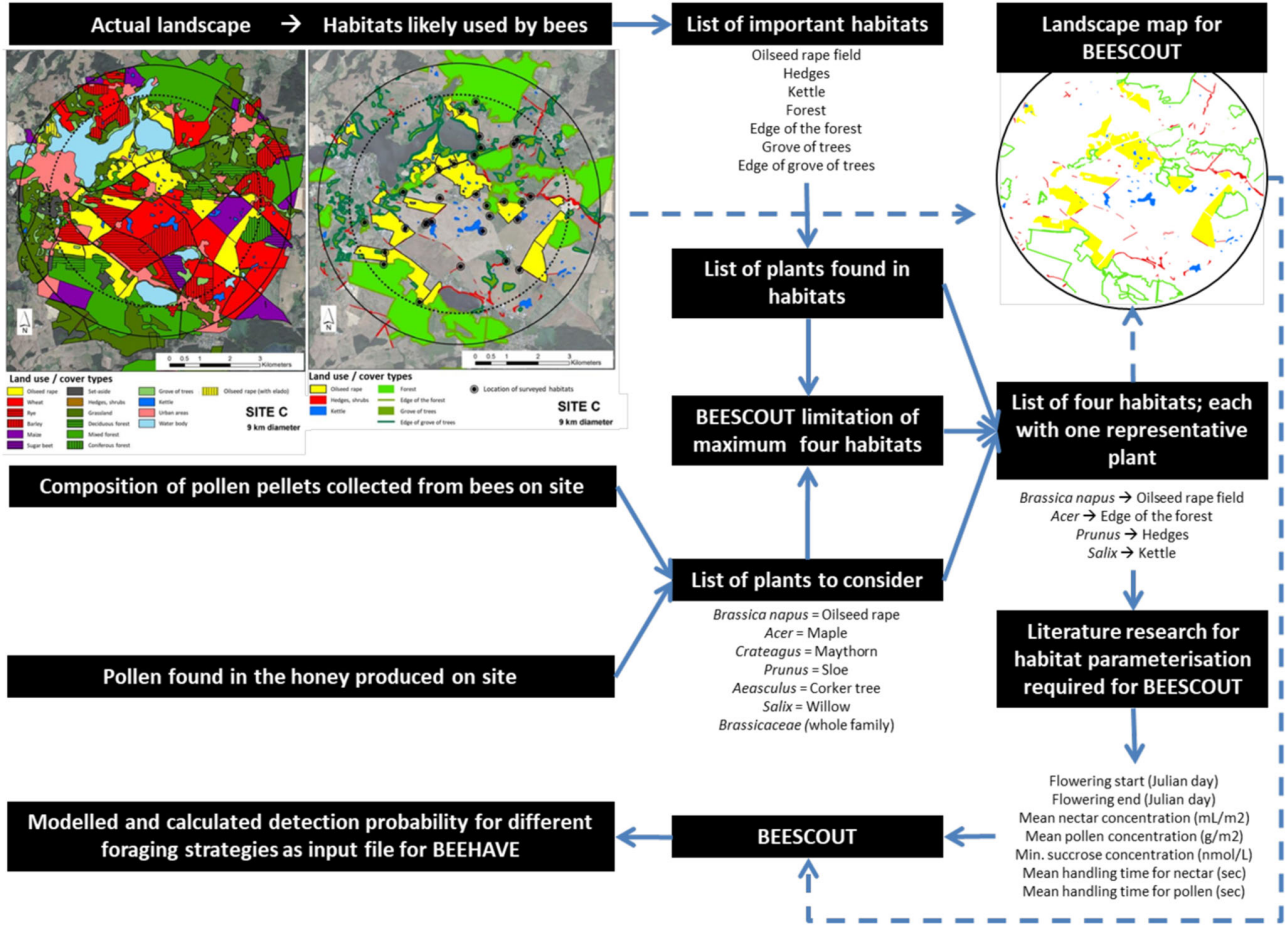
Schematic description of the landscape and habitat characterization from empirical data for landscape L1 of the field study and the wider literature for creation of bee foraging maps using BEESCOUT.

An analysis of the landscape around the hives was part of the field study (Heimbach et al. 2016). Although hives were located at the exposure site (L1), 17 different habitats were found in the 9‐km‐diameter landscape. Of these initial habitat types, 7 were categorized as being potentially important for honey bees because it was likely that foragers would be able to find and gather nectar and pollen within them. At the time of the experiment these 7 habitat types were investigated further at different locations within the 9‐km‐diameter landscape. This investigation included the observation on what flowering plants were found within the habitat types and at what density they were available. Furthermore, pollen pellets collected and honey produced from colonies placed in that landscape were analyzed for pollen composition to gather information on what plants were actually used as a food source. This information, combined with literature information (i.e., the nectar, pollen, and sugar content of those plants found to be most likely important for bee foraging), was used to identify and characterize the 4 possible habitats within BEESCOUT. An overview of the different information used and its interlinkage is described in Figure 1. Less detail for the landscape characterization of the relocation site (L2) was gathered in the field study. Therefore, the characterization of L2 for BEESCOUT modeling more intensively relied on the open literature. Additional information on the habitat characterization can be found in Supplemental Data, Tables S4 and S5. Within BEESCOUT it is currently not possible to change the landscape within a single run of the model to account for relocation of a beehive. Thus, we ran BEESCOUT individually for L1 and L2 and subsequently combined the data for the simulation of the whole field study with BEEHAVE.

### Landscape characterization strategies tested

Overall, we tested 6 different landscape characterization strategies using the information of the field study and BEESCOUT. The resulting food availability over time was then used as an input for BEEHAVE to simulate the field studies and compare observations and predictions with each other, focusing on the overall temporal agreement of bee colony development. For the first 2 strategies (“L1 short OSR” and “L1 long OSR”), we used the landscape information of L1 only and left the simulations running until the end of the field study, simulating that there has not been a relocation of the hives from L1 to L2 after 4 wk. The only difference between these 2 strategies was the duration of the oilseed rape flowering period. Very different information on the length of oilseed rape flowering was found in the literature. The same method of ignoring the relocation of the hives was conducted in strategies 3 and 4 (“L2 short OSR” and “L2 long OSR”), with the difference that here only L2 was used. The last 2 strategies tested were a mix of the other strategies. Here, a relocation of the hives from L1 to L2 after 4 wk was considered. This was realized by combining the BEESCOUT outputs “L1 short OSR” and “L2 short OSR” and “L1 long OSR” and “L2 long OSR” manually to create a combination of the landscapes with either short or prolonged oilseed rape flowering (“L1 + 2 short OSR” and “L1 + 2 long OSR”). To do this, the BEESCOUT outputs were combined after setting the food availability and detection probability of all food patches to zero from the day of hive relocation onward for L1 and for all food patches until the day of hive relocation for L2.

### BEESCOUT simulations

**Figure 2:**
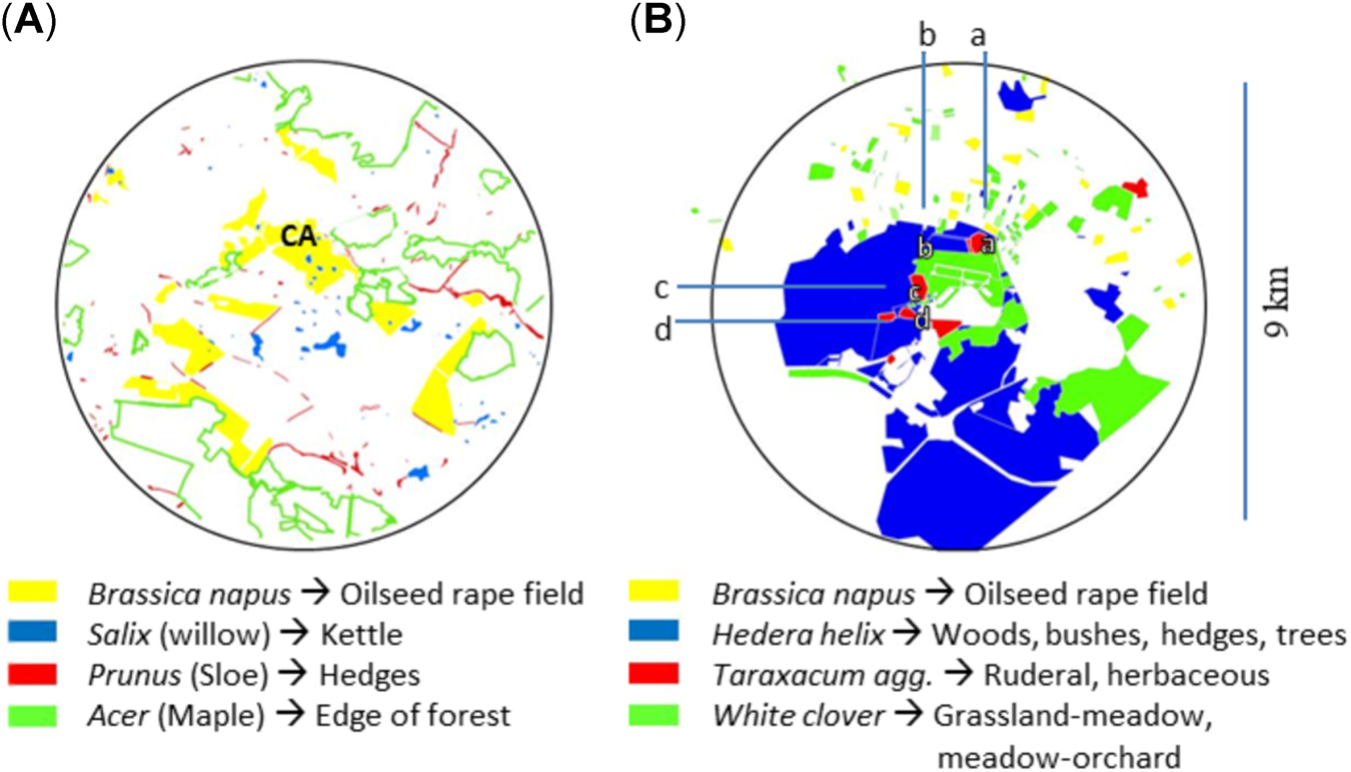
Overview of habitat characteristics for the landscapes L1 (**A**) and L2 (**B**) used in BEESCOUT. Both landscapes were used for BEEHAVE simulations indicated with L1 + 2. Letters inside the maps indicate the hive location(s) in the landscape(s). CA = control hive A.

We used the maps for L1 and L2 (Figure 2), the information on nectar and pollen availability for each habitat within the landscape specified in Table 2 (detailed information on the origin of these data is given in Supplemental Data, Table S2), and the information on the actual position of the hives to run the BEESCOUT model. BEESCOUT converts these inputs into a table useful for BEEHAVE. This table includes information on the distance of a food source to the hive, its nectar and pollen availability, and a detection probability for the foraging bees to detect this habitat as a food source. Model runs were conducted for L1 once (because in the present study data all 8 hives were jointly located) for each of the scouting modes “colony,” “mixed individual,” and “random location” with a random trip duration. The 3 scouting modes were chosen because they represent steep, medium steep, and shallow declines in the generated detection probability of a habitat for the bees in relation to the distance to the hive (Becher et al. 2016). Thus, 2 extreme cases and an intermediate one of the foraging range around the hive that bees are scouting for foraging grounds were covered. The “colony” search mode is described as a strategy a bee would follow if placed in an unknown landscape with no information from other bees. The maximum distance a bee travels within this search mode is 681 ± 54 m. The “mixed individual” search mode is a random selection of any search model that does not include a communication step between bees. Here, bees can travel 2350 ± 57 m away from the hive. The “random location” search mode is described as reflecting the foraging behavior of an established colony where exploration of the complete potential foraging area with appearing and disappearing food sources is present. In this case, maximum travel distance is determined by the user input of the maximum foraging range. More detailed information on the search modes can be found in the literature (Becher et al. 2016). The same runs were conducted for L2. The only difference is that this landscape was modeled 4 times for each of the 3 searching modes because the 8 hives selected were relocated to 4 different locations (a–d) within L2 (Figure 2). All other parameters and interface settings were kept at the default BEESCOUT model settings.

**Table 2:** BEESCOUT input used for the characterization of the 4 habitats in L1 and L2

	Habitat represented in the landscape	Representative pant	Latin name	Flowering start (day)^a^	Flowering end (day) ^a^	Mean nectar conc. (mL/m^2^)	Mean pollen conc. (g/m^2^)	Min. sucrose conc. (mol/L)	Mean handling time nectar (s)	Mean handling time pollen (s)
L1	n.a.	Willow male	*Salix* spp.	61	121	1.67	8.52	1.95	150 000	8826
	n.a.	Willow female	*Salix* spp.	61	121	2.01	0.00	0.20	125 000	n.a.
	Kettle	Willow average	*Salix* spp.	61	121	1.84	8.52	1.08	137 500	8826
	Hedges	Sloe	*Prunus spinosa*	61	152	0.43	0.30	0.58	3750	1607
	Edge of forests	European maple	*Acer platanoides*	92	152	3.41	20.44	1.75	1667	83
L1 and L2	Oilseed rape field	Oilseed rape (short)	*Brassica napus*	114	136	0.30	0.13	1.30	320	221
	Oilseed rape field	Oilseed rape (long)	*Brassica napus*	91	181	0.30	0.13	1.30	320	221
L2	Ruderal, herbaceous	Dandelion	*Taraxacum agg*.	1	364	0.37	10.64	0.409	36 232	375
	Grassland‐meadow and meadow‐orchards	White clover	*Trifolium repens*	140	242	0.049	0.0094	1.08	1630	2574
	Woods, bushes, hedges and trees	Ivy	*Hedera helix*	214	335	0.014	0.44	1.43	120 482	1165

^a^ Day represented as Julian day.

n.a. = not applicable.

### BEEHAVE simulations

The overall interface settings that were kept constant for all BEEHAVE simulations conducted were 1) simulation start on Julian day 116 (26 April); 2) no varroa at the beginning of the run but allowed mite reinfestation at a rate of 0.21/d (derived from the field data) and using the mite reproduction model “Martin”; 3) no varroa treatment (see Supplemental Data for more detail); 4) honey extraction from the hives on Julian days 151 and 201 assuming a constant amount of honey (10 kg) to remain in the hive; 5) feeding of the hives on Julian days 146, 196, 220, and 227 with 2.5, 5, 7, and 7 kg, respectively (as was conducted in the field study); 6) an age of the queen of 308 d; 7) a maximum number of brood cells available in the hive of 109 520; and 8) inactivity of the “StopDead” function. Further details on the starting conditions for the runs can be found in Supplemental Data, Table S3. All simulations were run with the stochasticity of the model activated. A total of 200 runs per simulation were conducted, and all results are presented as the average value of these 200 runs and the corresponding 95% tolerance interval, that is, average ± (1.96 × standard deviation).

First, we simulated the average colony dynamics for all 6 different landscape characterization strategies used in BEESCOUT (“L1 short OSR,” “L1 long OSR,” “L2 short OSR,” “L2 long OSR,” “L1 + 2 short OSR,” and “L1 + 2 long OSR”) using the average starting conditions of the hives given in Table 1. These runs were conducted to investigate how landscape characterization influences simulation outputs and what strategy performs best for good agreement between simulated and observed colony development. For these simulations, the BEESCOUT outputs for hive location b in landscape L2 were used to create the input files for “L1 + 2 short OSR” and “L1 + 2 long OSR.”

The same simulations were conducted for each hive separately, using the hives' own starting conditions (Table 1) and the hives' own BEESCOUT outputs for their individual location profile. These simulations were conducted to investigate whether 1) the position of a hive within L2 influences the population dynamics and 2) the different starting conditions of individual hives are the driving or influencing factor for variability between hives observed in the field study.

We conducted the model runs using landscapes L1, L2, and L1 + L2 to investigate how detailed the landscape characterization needs to be to allow simulation of the patterns of the colony dynamics observed in the field study. We are aware that only using L1 or L2 does not truly reflect the situation in the field study. However, choosing these settings in comparison will provide vital information on how much effort needs to be put toward the landscape characterization for future model validation studies, landscape investigations in field studies, and creation of model scenarios for risk‐assessment purposes. As mentioned, the field study used in the present study (but also most other field studies currently available) did not conduct a landscape investigation, honey analysis, and pollen analysis during the second phase (postexposure phase). Thus, the landscape characterization for this location for BEESCOUT simulations relies mainly on open literature and expert judgment, not reflecting the same depth of information as for the first phase of the study (exposure phase). Furthermore, currently there is no opportunity to simulate a relocation of a hive in the BEEHAVE model in a straightforward way. Thus, BEEHAVE‐Weather files and BEESCOUT output files need to be merged to reflect the relocation of a hive. Investigating whether implementation of a hive relocation might be required is another reason we ran BEEHAVE with the different types of landscape characterization.

All mentioned BEEHAVE simulations were conducted twice. BEESCOUT produces 2 different detection probabilities for each patch: a modeled and a calculated detection probability. We investigated whether and, if so, how intensively these different detection probabilities affect BEEHAVE outputs. Several simulations were additionally conducted 3 times because we have 3 BEESCOUT outputs representing the landscape description for BEEHAVE under the search modes “colony,” “mixed individual,” and “random location.”

## RESULTS AND DISCUSSION

The limitations of defining a very detailed nectar and pollen availability in BEESCOUT still allow the simulation of bee colony dynamics in a heterogeneous landscape that are in good agreement with measured data. We define “good agreement” between study data and BEEHAVE outputs as visually showing similar temporal patterns and similar abundance of individuals from the different cohorts (adult bees, eggs, larvae, and pupae).

**Figure 3:**
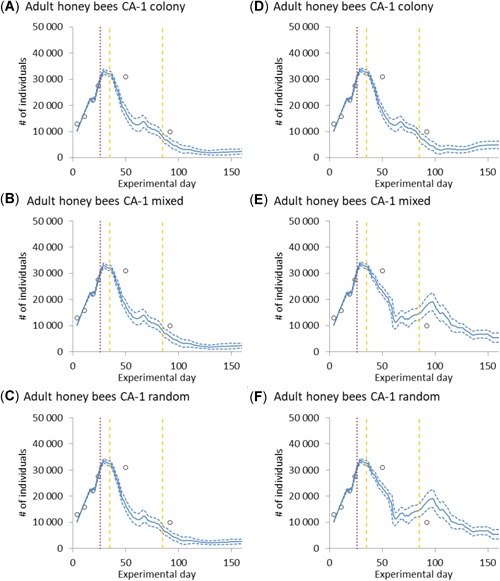
Measured and simulated number of adult bees of control colony A‐1 (CA‐1) over time. Simulations were conducted using the individual colony starting conditions of hive CA‐1, the “colony,” “mixed individual,” or “random location” scouting strategy for both the short (**A**–**C**) and long (**D**–**F**) oilseed rape flowering period (*n = *200). Red dotted line indicates movement of hives between landscapes L1 and L2. Orange dotted lines indicate the time honey was extracted from the hives.

There are BEESCOUT settings which are related to foraging (i.e., scouting strategy and flowering duration in BEESCOUT) that can strongly influence the simulated colony dynamics. Figure 3 illustrates that both the scouting strategy used in BEESCOUT and the duration of flowering of the most melliferous plant available (here oilseed rape) influence the simulated number of adult bees over time. Differences in food availability derive from the interplay of maximum foraging distance and duration of oilseed rape flowering and can reach such high levels that colonies can collapse. Colony collapse in this case is an absence of or very limited further brood production that results in an almost steady decline in the number of adult bees (an illustration for this is provided in Supplemental Data, Figure S1). In the present study data, colonies collapse in all simulations when using the short oilseed rape flowering period and when using the scouting strategy “colony” in combination with long oilseed rape flowering (Figure 3A–D). Using any of the other scouting strategies tested and the prolonged oilseed rape flowering prevented colony collapse in the simulations (Figure 3E and F).

**Figure 4:**
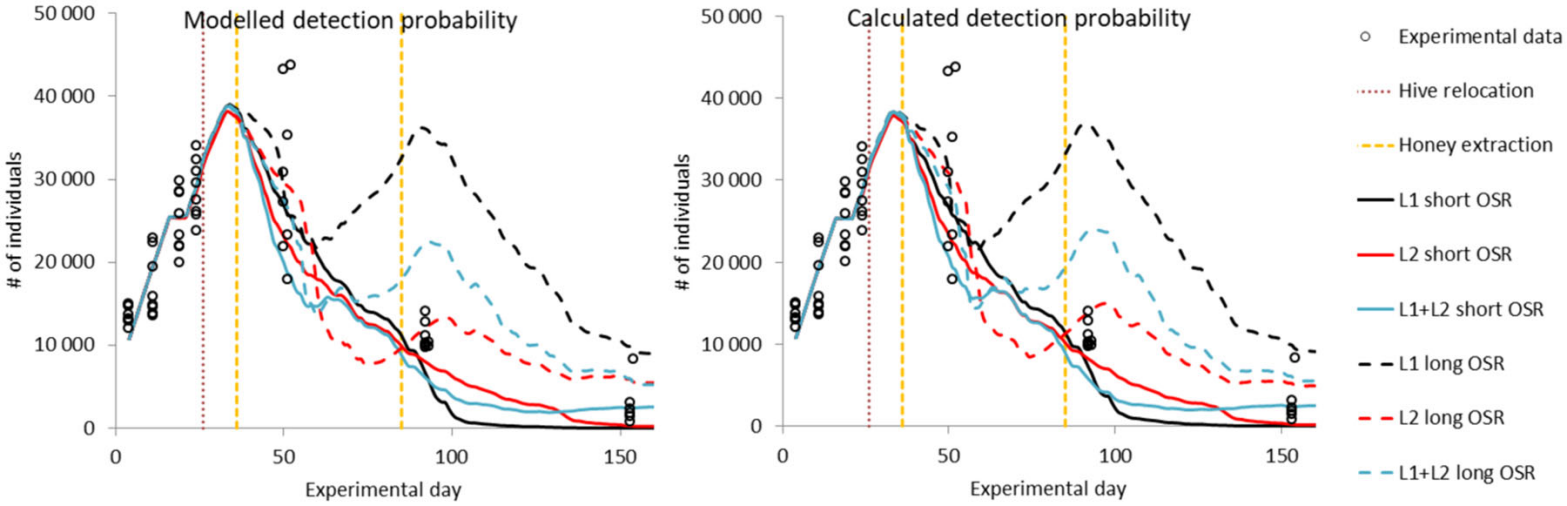
Measured and simulated number of adult bees over time. Experimental data illustrate the individual replicate colonies. Simulations for the different landscape characterization strategies are illustrated as average (*n* = 200). Simulations were conducted using the average colony starting conditions, the “random location” scouting strategy, and either the modeled habitat detection probability (left) or the calculated detection probability (right). Red dotted line indicates movement of hives between landscapes L1 and L2. Orange dotted lines indicate the time honey was extracted from the hives. OSR = oilseed rape.

When focusing on the landscape characterization in terms of using landscape L1 or L2 only or in combination (L1 + L2) to simulate the colony dynamics, there is generally no difference in the simulated number of adult bees for the initial experimental phase. Differences start to occur at approximately the time when honey was extracted the first time (Figure 4).

The absence of differences between simulations until first honey extraction from the hives, in combination with the fact that we are predicting colony development rather well within the initial phase, illustrates that colony development in that phase of the experiment is predominantly driven by the initial hive conditions and not by the accuracy of landscape characterization and food availability in the surrounding landscape. Brood already in the hive and resources accumulated prior to the start of the experiment determine the development of the colony in the first period. However, food available for gathering during that phase influences the long‐term development of the colony because resources in the hive are affected, but this impact only manifests itself in the observed endpoints after some time delay.

Again, there is no substantial impact of the landscape combination used for the simulations when oilseed rape flowering was short because all colonies collapsed (Figure 4, solid lines). However, a substantial impact of the landscape occurred when considering long oilseed rape presence, indicating that a simplification of the landscape characterization for simulation of field studies by neglecting the relocation of the hives is a nonacceptable assumption.

Simulation results and their comparison to field observations are detailed in the Supplemental Data, showing population development when separating the bees into different cohorts (eggs, larvae, pupae, adults) as well as the production of extractable honey and the development of varroa mites in the hive (illustrated as mite fall per day). Figures in the Supplemental Data also illustrate the 95% tolerance intervals for all simulations. Supplemental Data, Figure S2, shows that, overall, the use of the landscape characterization closest to the situation in the field study produces the best simulation results in comparison with measured data, even though Figure 4 suggests that the use of only L2 with long oilseed rape flowering would be sufficient. This implies that the landscape in the postexposure phase, which is not well investigated in field studies, is much more important in the simulation of the colony strength than the normally well‐investigated exposure landscape.

BEESCOUT settings that influence the use of the landscape characterization (i.e., the use of the modeled and calculated detection probability) do not in the illustrated cases have any impact on the colony dynamics (Figure 4). Further simulations conducted using higher nectar availability of *Taraxacum* agg. (i.e., 19.5 rather than 0.37 mL/m^2^) that provokes *Taraxacum agg*. to be the most melliferous plant within the landscape showed that the use of the modeled or calculated detection probability can cause significant changes to colony development (Supplemental Data, Figure S3).

One assumption used for the simulations presented in Figure 4 is that for the simulations with L2 (red line) all 8 hives were placed together at one of the 4 hive locations (location b illustrated in Figure 2). Best agreement between simulation and study data was found when simulations were conducted accounting for all hive‐specific conditions, using the scouting strategy that allowed full exploration of the provided landscape (i.e., “random location”). See Supplemental Data, Figure S4, for the total abundance of adult bees over time and Figure S5 for all endpoints observed.

Exceptions of good agreement are the underpredictions of the first honey extraction that took place shortly after relocation of the hives, the overprediction of the number of adult bees for the second‐to‐last observation time point, and the underprediction of mite fall (Supplemental Data, Figure S5). We observed a constant and over time increasing underprediction of the mite population that manifested a 10‐ to 30‐fold underprediction of the daily mite fall. This in turn causes an overprediction of adult bees because pupa and adult bee mortality from varroa infestation was simulated to be too low. In addition, Simone‐Finstrom et al. (2016) demonstrated that bees from hives relocated during summer for pollination management show a decrease in survival probability manifested in a shorter life span. Such an increased stress‐related mortality of bees is currently not included in the BEEHAVE model. Further investigation is needed on whether such an additional stress factor needs to be included in the model to fulfill the model's purpose to serve as a tool for the modeling of the effects of multiple stressors on bees within the risk‐assessment scheme.

**Figure 5:**
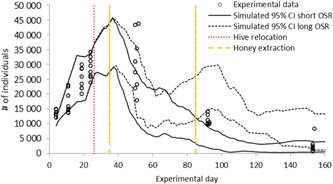
Measured and simulated number of adult bees over time. Experimental data (points, 8 replicates) illustrate the individual hive. Simulations for each of the 8 hives using the landscape characterization strategy L1 + L2 with the hive locations a through d within landscape L2 are illustrated as the minimum and maximum 95% tolerance interval from all 8 individual hives for simulations (*n* = 200) with short (black lines) and long (black dotted lines) oilseed rape flowering periods. Simulations were conducted using the individual colony starting conditions, the individual hive locations in L1 and L2, the “random location” scouting strategy, and the modeled habitat detection probability. Red dotted line indicates movement of hives between the 2 locations. Orange dotted lines indicates the time honey was extracted from the hives. CI = confidence interval; OSR = oilseed rape.

In all but one case (day 50) the variability among simulated average number of adult bees is similar in magnitude to the variability in the observed field data for each day and observed data are within the simulated 95% tolerance intervals (Figure 5). That our simulations do not match the large variability in the measured data on day 50 is most likely driven by 2 circumstances. First, the model shows that in all replicates exactly at approximately this day a steep decline in the number of adult bees occurs. Thus, a small temporal shift in either the assessment in the field or the temporal accuracy in the model can have a large impact on the overlap of simulation and measurement. This is why we chose not to conduct a comparison of simulation and measurement via any statistical means. In addition, and most likely the factor that has a greater impact on the variability, is the fact that within the simulations we assume a fixed amount of honey that remains in the hive after the beekeeper extracted honey. Our assumption is that each hive has the exact same amount of honey (10 kg). This forced all simulations into the same direction and thus reduced the variability between colonies. In the field it is likely that each hive contained different amounts of resources after the beekeeper removed the upper supers for honey extraction.

### Future BEEHAVE evaluation and scenario development

The present study is a first step toward providing evidence that BEEHAVE represents the vital processes involved in the development of honey bee colonies, taking into account the heterogeneous landscape around the hive and varroa mite infestation. Overall, the model predicts the beehive dynamics well when focusing on the temporal pattern of the total number of adult bees, the total number of offspring in the hive, and the production of drones. The present study underpins the results of the EFSA evaluation of the BEEHAVE model (European Food Safety Authority 2015) that the most important in‐hive dynamics are represented and correctly implemented in the model with empirical evidence. Further evaluation studies similar to the present study are needed to gain confidence that the model does perform well in simulating colony dynamics in agreement with a wider array of empirical studies representing different weather, landscape, and disease conditions within one European zone and across different zones. Such evaluation studies are needed to gain confidance in the use of BEEHAVE and BEESCOUT as suitable tools for higher‐tier environmental risk assessment. Another vital part for BEEHAVE enhancement, to allow its use for higher‐tier environmental risk assessment of plant protection products, is the comprehensive implementation of mechanisitc exposure and effect modeling. Furthermore, it is advisable to also conduct investigations on whether the use of BEESCOUT with its limitations and its high demand of information on pollen, nectar, sugar content, and handling time of plants is the way forward to finding a balance between data demand for parameterization and simulation accuracy. However, an alternative to the use of BEESCOUT is currently not available. There have been recent developments in the assessment of food availability for bees that are more realistic and use much more complex landscapes in terms of nectar (Baude et al. 2016) and nectar and pollen availability (Hicks et al. 2016) that might be methods to improve the representation of landscapes for BEEHAVE modeling purposes. These methods, however, need further investigation on whether there are ways of converting the calculated potential nectar and pollen availability into actual availability in relation to the location of a hive, the scouting practices of bees, and the uncertainty in the results from the advanced methods. It is unclear whether the advanced methods can provide more robust and reliable information for BEEHAVE than BEESCOUT currently does. In addition, further evaluation studies should consider the overwintering success of colonies as a vital endpoint for comparison with field data because this endpoint, next to colony strength, is considered an important measure for the higher‐tier risk assessment for bees.

The EFSA evaluation of the BEEHAVE model (European Food Safety Authority 2015) mentions that the model is in its current state not ready to be used for risk‐assessment purposes because, among other things, there is currently only one landscape scenario included that is ready to be used (i.e., readily parameterized) and thus could serve as a standard scenario for model predictions. The number of scenarios certainly needs to be increased. To do this, scenarios need to be defined in regard to their overall representation of landscapes, climates, and events (stress combinations) that are realistic and cover the full range of European conditions.

### Recommendations for the design of field studies

It is clear that data gathered in field studies are not optimal data sets for model use and model evaluation because these studies are tailored toward a specific question and currently do not have a standard protocol to follow. There are, however, certain things that could be considered in the development of future field studies that would benefit the field study and its interpretation and the use of the study for modeling purposes. We show that BEEHAVE can capture the variability within measured data and simultaneously that the different starting conditions of a hive at the beginning of the experiment greatly influence both the subsequent colony dynamics and the variability among replicates. Thus, the model can be used to investigate whether the variability among the replicates of the control colonies observed in a field study is solely driven by the differences between the colonies at the start of the experiment or whether other stressors contribute to the variability and therefore should be considered in the analysis of the field data. For such an application of the model, the starting conditions of the hives need to be recorded. Starting conditions in that sense means the recording of bee abundance (including the separation into different life stages and castes), honey and pollen stores, the exact availability of brood cells, and the age of the queen (in days or weeks rather than years). Furthermore, information for the characterization of the landscape at the time of the experiment is needed. Overall, we show that impacts driven by foraging do not manifest themselves as a deviation between simulated and measured colony dynamics immediately because the colony starting conditions are driving the simulated colony dynamics almost entirely in the first weeks. This impact then decreases over time and is increasingly substituted by the impact of the foraging activity, which, driven by the surrounding landscape, influences the nectar and pollen resources of the colony and ultimately drives the colony dynamics. The frequency and intensity of data gathered during field studies vary between studies and within studies over time. Common among studies is that data availability for hive observations and landscape characterizations is greater for the proportionally short exposure phase in comparison to the postexposure phase. A redistribution of experimental efforts allocated to a field study toward a more equal bee colony and landscape investigation throughout the experiment, rather than a bias toward the actual exposure phase, will allow the field study to be used for model evaluation. Particularly the often missing or very limited information of the landscape the hives are relocated to after the exposure phase of the experiment makes model parameterization for potential foraging activity within that phase difficult and inacurate. Besides alterations in the study plan toward unbiased temporality of data gathering, documentation of procedures and results at the first glance not necessary for the analysis of a field study would greatly support modeling efforts. Any beekeeper activity should be described in detail and underpinned with qualitative data so that these can be implemented in the model. For example, when colonies are fed it is vital to know not only the total amount of food provided per colony during autumn and winter but also how much food was provided on what day. Similarly, when honey is extracted from the hive it is important for modeling to know when and how much honey was extracted and how much was left in the hive.

## Supplemental Data

The Supplemental Data are available on the Wiley Online Library at DOI: https://doi.org/10.1002/etc.4547

## Supporting information

This article contains online‐only Supplemental Data.

Supporting information.

## Data Availability

All data generated and analyzed during the present study are included in this published article or the Supplemental Data or can be made available on request (annika.agatz@ibacon.com). All alterations made to the original BEEHAVE version from 2016 are documented as code sections in the Supplemental Data.
