# Supplementary material for: An Evaluation of the BEEHAVE Model Using Honey Bee Field Study Data: Insights and Recommendations
Source: Environ Toxicol Chem. 2019 Sep 24;38(11):2535–45. doi: 10.1002/etc.4547 (PMC6856857; doi:10.1002/etc.4547)
Supplement: Supplementary file 1 — Supporting information. [file ETC-38-2535-s001.docx]

# SUPPLEMENTAL DATA

**Title:** BEEHAVE evaluation on honey bee field studies: insights and recommendations

Number of tables: 5

Number of Figures: 5

Additional information for Table 1

Additional information on habitat characterisation

Additional information on varroa treatment during the field study

NetLogo code sequences for the BEEHAVE alterations made

Table S1: List of additions made to the input and output of the original BEEHAVE model to implement the option to run specific field experiments at a flexible starting day in a year with flexible hive starting conditions.

| **Type** | **Name** | **Description** | **Unit** |
| --- | --- | --- | --- |
| Switch | exp_input_initial_conditions | option to provide specific starting conditions for an experiment | [ ] |
|  | exp_feeding-shedule | option to add specific hive feeding instructions for an experiment | [ ] |
|  | exp_Honey-harvest | option to add specific days where honey is harvested | [ ] |
| Inputs | exp_StartDay | Resets the timing of the model to start at any given Julian day | [doy] |
|  | exp_init_IHbees | Sets the number of adult worker bees at the start of the run | [individuals] |
|  | exp_init_eggs | Sets the number of worker eggs at the start of the run | [individuals] |
|  | exp_init_eggs_drone | Sets the number of drone eggs at the start of the run | [individuals] |
|  | exp_init_honey | Sets the honey stored in the hive at the start of the run | [kg] |
|  | exp_init_pupae | Sets the number of worker pupa at the start of the run | [individuals] |
|  | exp_init_larvae | Sets the number of worker larva at the start of the run | [individuals] |
|  | exp_init_larvae_drone | Sets the number of drone larva at the start of the run | [individuals] |
|  | exp_init_pollen | Sets the pollen store in the hive at the start of the run | [kg] |
|  | exp_Age-of-Queen | Sets the age of the queen at the starting doy of the run | [days] |
|  | exp_init_pupae_drone | Sets the number of drone pupa at the start of the run | [individuals] |
|  | exp_feeding-day1 | Day where food is added | [doy] |
|  | exp_feeding-day2 | Day where food is added | [doy] |
|  | exp_feeding-day3 | Day where food is added | [doy] |
|  | exp_feeding-day4 | Day where food is added | [doy] |
|  | exp_added-fondant1 | Amount of food added | [kg] |
|  | exp_added-fondant2 | Amount of food added | [kg] |
|  | exp_added-fondant3 | Amount of food added | [kg] |
|  | exp_added-fondant4 | Amount of food added | [kg] |
|  | exp_harvest-day1 | Day where honey is harvested | [doy] |
|  | exp_harvest-day2 | Day where honey is harvested | [doy] |
|  | exp_harvest-day3 | Day where honey is harvested | [doy] |
|  | exp_harvest-day4 | Day where honey is harvested | [doy] |
|  | exp_remaining-honey1 | honey remaining in the hive after honey harvest | [kg] |
|  | exp_remaining-honey2 | honey remaining in the hive after honey harvest | [kg] |
|  | exp_remaining-honey3 | honey remaining in the hive after honey harvest | [kg] |
|  | exp_remaining-honey4 | honey remaining in the hive after honey harvest | [kg] |
| Diagram | A diagram option "AGA own" was added that produces all  outputs to be directly comparable to assessments made in the field | | [ ] |

Doy = day of the year

Table S2: Parameter values used for the calculation of BEESCOUT input parameters (presented in Table 1) for the landscape characterisation. Numbers in brackets indicate the reference that either states the value given here directly or provided information to calculate the given value.

| **Crop** | **Oilseed rape** | **White clover** | **Willow (male)** | **Willow (female)** | **Sloe** | **European maple** | **Maythorn** | | **Dandelion** | | | **Ivy** | |
| --- | --- | --- | --- | --- | --- | --- | --- | --- | --- | --- | --- | --- | --- |
| Latin name | *Brassica napus* | *Trifolium repens* | *Salix caprea* | *Salix caprea* | *Prunus spinosa* | *Acer platanoides* | *Crateagus monogyna* | | *Taraxacum agg.* | | *Hedera helix* | |  |
| Flowering period | April-May | May-October | March-April | March-April | March-May | April-May | May-June | | All year | | | August-November | |
|  | (1) | (1) | (2) | (2) | (2) | (2) | (2) | | | (3) | | (3) | |
| Mean Flowering (days) | 22^a^, 90^b^ | 102 | 60 | 60 | 91 | 60 | 60 | | 365 | | | 121 | |
|  | (1)^a^, (3)^b^ | (1) | (2) | (2) | (2) | (2) | (2) | | | (3) | | (3) | |
| Min Nectar concentration (µl/flower/day) | 0.35 | 0.02 | 0.005 | 0.005 | 0.15 | 0.8 | 0.1 | | n.a. | | | n.a. | |
|  | (1) | (1) | (4) | (4) | (5) | (6) | (5) | | |  | |  |  |
| Mean Nectar concentration (µl/flower/day) | 0.55 | 0.1 | 0.01 | 0.012 | 0.4 | 0.9 | 0.36 | | 0.041 | | | 0.012 | |
|  | (1) | (1) | (4) | (4) | (5) | (6) | (5) | | | (3)^c^ | | (3)^c^ | |
| Max Nectar concentration (µl/flower/day) | 0.82 | 0.18 | 0.017 | 0.027 | 0.8 | 1 | 0.7 | | n.a. | | | n.a. | |
|  | (1) | (1) | (4) | (4) | (5) | (6) | (5) | | |  | |  | |
| Min Pollen concentration (mg/flower/day) | 0.187 | n.a. | 0.039 | 0.039 | n.a. | 2.29 | n.a. | | n.a. | | | 0.359 | |
|  | (1) |  | (7) | (7) |  | (7) |  | |  | | | (8) | |
| Mean Pollen concentration (mg/flower/day) | 0.239 | 0.019 | 0.0509 | 0.0509 | 0.28 | 5.39 | 0.33 | | 1.2 | | | 0.386 | |
|  | (1) | (1) | (7) | (7) | (9) | (7) | (9) | | (10) | | | (8) | |
| Max Pollen concentration (mg/flower/day) | 0.292 | n.a. | 0.067 | 0.067 | n.a. | 11.4 | n.a. | | n.a. | | | 0.412 | |
|  | (1) |  | (7) | (7) |  | (7) | |  |  | | | (8) | |
| Min sucrose concentration in nectar (%) | 44 | 37 | 66.7 | 7 | 20 | 60 | 40 | | 14 | | | 49 | |
|  | (1) | (1) | (4) | (4) | (5) | (6) | (5) | | (11) | | | (12) | |
| Max Sucrose concentration in nectar (%) | 59 | 65 | 98.1 | 52.1 | 60 | 60 | 70 | | 55 | | | 49 | |
|  | (1) | (1) | (4) | (4) | (5) | (6) | (5) | | (11) | | | (12) | |
| Min Number of flowers (/m2/day) | 543 | 247 | 0 | 0 | 40.4 | 0 | 0 | | 1027 | | | 375 | |
|  | (1) | (1) | (2) | (2) | (2) | (2) | (2) | |  | | | (3) | |
| Max Number of flowers (/m2/day) | 1194 | 741 | 334178 | 334178 | 2134 | 7582 | 19003 | | 16705 | | | 1912 | |
|  | (1) | (1) | (2) | (2) | (2) | (2) | (2) | | (3) | | | (3) | |
| Min visiting time per flower (s) | 3.52 | 2.64 | 3.4 | 3.4 | 3.4 | 3.4 | 3.4 | | 3.4 | | | 3.4 | |
|  | (1) | (1) | (13) | (13) | (13) | (13) | (13) | | (13) | | | (13) | |
| Mean visiting time per flower (s) | 3.52 | 3.26 | 30 | 30 | 30 | 30 | 30 | | 30 | | | 30 | |
|  | (1) | (1) | (13) | (13) | (13) | (13) | (13) | | (13) | | | (13) | |

n.a.: not available; ^a^ for the short oilseed rape flowering period; ^b^ for the long oilseed rape flowering period; ^c^ Literature data used to calculate this value.

**Additional information for Table 1**

On the first assessment day during the field study hives were analysed *via* the picture analysis method (analysis of comp cells with the Software HoneybeeComplete from digital Photos). All cells that were not analysed to be filled with brood or were empty were judged to be filled with honey, nectar or pollen. Whilst nectar/pollen cells were specifically analysed capped honey cells were not counted. Nonetheless, the number of cells filled with honey could be calculated. For this we assumed that each comp had a fixed number of cells (2738; <https://sites.google.com/site/imkervereinladenburg/wissenswertes/die-wabe-und-das-wachs>; last accessed 27.09.2018) and the difference between all specifically analysed cells (nectar/pollen, empty, eggs, larva, pupa) and the number of cells available were filled with honey. We furthermore assumed that each cell has a volume of 0.36 ml and contains either 500 mg honey or 230 mg pollen (14).

BEEHAVE does not make a separation between honey and nectar. So we also assumed no difference in our calculation and added the counted nectar/pollen filled cells to the calculated honey. Prior this, we had to deduct a part of the nectar/pollen cells to specify how many of these were filled with nectar and how many were filled with pollen. We decided to use a factor of 6 between the proportion of nectar and pollen filled cells for the calculation according to values found in the literature (14).

Additional information on the data on the nectar available per flower and day used from the literature provided in Table S2 marked with ^c^, were subject to a calculation because only information on the nectar sugar mass per flower and day was found. The numbers from the literature were used to calculate the nectar volume per flower and day accounting for the sugar content of the nectar for the particular plant, that was found in another literature (and is also provided in Table S2).

**Table S3: Additional information on the BEEHAVE model settings in the interface**

| **Input variable** | **Input** |
| --- | --- |
| EggLaying_IH | true |
| ReadBeeMappFile | false |
| ReadInfile | true |
| Weather | Weather File |
| INPUT_FILE | Landscape file created in BEESCOUT |
| WeatherFile | Weather file created with BEEHAVE-weather |
| RAND_SEED | 0 |
| exp_StartDay | 116 |
| X_Days | 150 |
| N_INITIAL_MITES_HEALTHY | 0 |
| N_INITIAL_MITES_INFECTED | 0 |
| AllowReinfestation | true |
| MiteReinfestation | 0.21 |
| VarroaTreatment | false |
| HoneyHarvesting | false |
| MergeWeakColonies | false |
| FeedBees | false |
| AddPollen | false |
| HoneyIdeal | false |
| PollenIdeal | false |
| Swarming | No swarming |
| MAX_BROODCELLS | 109520 |
| SeasonalFoodFlow | true |
| ConstantHandlingTime | false |
| TIME_NECTAR_GATHERING | 483 |
| TIME_POLLEN_GATHERING | 600 |
| SHIFT_R | 30 |
| SHIFT_G | -40 |
| AlwaysDance | false |
| Experiment | none |
| MAX_km_PER_DAY | 7299 |
| stopDead | false |
| ProbLazinessWinterbees | 0 |
| SQUADRON_SIZE | 100 |
| modelledInsteadCalcDetectProb | true |
| ShowAllPlots | true |
| stopDead | true |
| HiveType | Dadant |
| FrameType | Standard brood/deep frame |
| exp_input_initial_conditions | true |
| exp_Age-of-Queen | 308 |
| exp_init_IHbees | See Table 1 for details |
| exp_init_pupae | See Table 1 for details |
| exp_init_larvae | See Table 1 for details |
| exp_init_eggs | See Table 1 for details |
| exp_init_pupae_drone | See Table 1 for details |
| exp_init_larvae_drone | See Table 1 for details |
| exp_init_eggs_drone | See Table 1 for details |
| exp_init_pollen | See Table 1 for details |
| exp_init_honey | See Table 1 for details |
| exp_feeding-shedule | true |
| exp_feeding-day1 | 146 |
| exp_feeding-day2 | 196 |
| exp_feeding-day3 | 220 |
| exp_feeding-day4 | 227 |
| exp_added-fondant1 | 2.5 |
| exp_added-fondant2 | 5 |
| exp_added-fondant3 | 7 |
| exp_added-fondant4 | 7 |
| exp_Honey-harvest | true |
| exp_harvest-day1 | 151 |
| exp_harvest-day2 | 201 |
| exp_remaining-honey1 | 10 |
| exp_remaining-honey2 | 10 |

**Additional information on habitat characterisation**

Table S4: Plants found in pollen pellets and honey while bees where in the landscape 1.

| Plant | Found in which hives? | Frequency of finding it |
| --- | --- | --- |
| *Brassica napus* | All | 50-90% in honey; more in late pollen pellet (day 19-23 than in early one (day 15)) |
| *Acer plantanoides* | Almost all. Tendency towards the control site compared to the treatment site. | Max. 10% in honey; between 5 and 80% in pollen pellets. More in early pellet analysis than in the late one. |
| *Aesculus hippocastanum* | ~50% of treatment sites distant from OSR fields for honey analysis. | 25 to 50 % of early pollen sample analysis between 5 and 50% of all pollen. |
| *Barbarea vulgaris and Isatis tinctoria (grouped as Brasicaceae)* | Only in treatment site. | In about 50% of the early pollen assessment, not found in the honey. |
| *Crataegus monogyna* | Almost all. Tendency towards the treatment site than the control site. | 5-45 % in the control sites and 2-25% in the treatment site. |
| *Prunus spinosa* | Almost all; no apparent difference between control and treatment site. | Not found in the pollen pellets but with up to 20% found in the honey. |
| *Salix alba, S. fragilis* | CA and little in CB for pollen pellets. All hives for honey analysis. Tendency towards more found in the control plot than in the treatment plot. | Between 10 and 30% in honey from control plot and 5 to 20% for treatment plots. |
| *Salix aurita* |  |  |
| *Salix caprea, S. daphnoides* |  |  |
| *Sorbus aucuparia* | CF, TB, TC, TE | Between 5 and 20% in early pollen analysis. Almost nothing in late pollen analysis. Not in honey. |
| *Sorbus intermedia* |  |  |
| *Taraxacum spec.* | One hive in CD and one in CF | 5 to 10% in early pollen analysis. |

Table S5: Documentation on the decision making process for plant allocation to available habitats in landscape 1 of the bee field study.

| Habitat type | Habitat important for bees according to habitat mapping | Plant allocated to in this habitat | Found in pollen and honey analysis | Decision made from pollen and honey analysis, and habitat observation | Decision made from pollen and honey analysis, and hive location | Reasoning for decision made | |
| --- | --- | --- | --- | --- | --- | --- | --- |
| Barley |  | ***Hordeum vulgare*** |  |  |  | **Arable crop** | |
| Sugar Beet |  | ***Beta vulgaris*** |  |  |  | **Arable crop** | |
| Maize |  | ***Zea mays*** |  |  |  | **Arable crop** | |
| Cultivated Grassland Meadow | X | *Taraxacum agg.* | X |  |  | According to the literature this plant can be very attractive to bees and can play a major role for colonies when abundant and is next to *Trifolium repens* one of the largest nectar provider in improved grassland (3). | |
| Kettle with Shrubs | X | *Salix* | X | X |  | Habitat mapping during exposure phase indicates that *S. alba, S. fragiles, S. auria, S. caprea,* and *S. daphnoides* were most frequently found in hedges, both in the control and in the treatment area. | |
| Wood |  | *Acer* | X |  | X | Overall there seems to be more acer in pollen pellets collected by hives in close approximation of this habitat. | |
| Oilseed Rape | **X** | ***Brassica napus*** | **X** | **X** |  | **Arable crop;** abundantly found in honey and pollen | |
| Rye |  | ***Secale cereale*** |  |  |  | **Arable crop** | |
| Set aside |  |  |  |  |  |  | |
| Urban Areas | X |  |  |  |  | This area was indicated to be important, but there is no further information on why and with what plant(s). | |
| Bushes Shrubs Hedges | X | *Prunus spinosa* | X | X |  | Was abundantly found in pollen and honey analysis. | |
| Sand-sedge |  |  |  |  |  |  | |
| Triticale |  | ***Triticosecale*** |  |  |  | **Arable crop** | |
| Wheat |  | ***Triticum aestivum*** |  |  |  | **Arable crop** | |
| Water | (X) | *NA* |  |  |  | Water should be included into the map. Bee flying behaviour is influences by open water, as stated in the BEEHABE and BEESCOUT description. | |
| Edge of grove of trees | X * | *Crataegus monogyna* | X |  | X | According to the hive locations, *Crateagus* content in the pollen and honey seems to be a correlation with % pollen and closeness to grove of trees. | |
| Grove of trees | X | *Aesculus hippocastanum* | (X) |  | X | Quite similar to *Crataegus monogyna* | |
|  | X | *Barbarea vulgaris* | (X) |  | X | The plant prefers fresh or moist places, on roadsides, along rivers, in arable land, wastelands and docklands, or on the slopes and in ditches, at an altitude of 0–2,500 m (0–8,202 ft) above sea level (Wikipedia). | |
|  | X | *Sorbus* | (X) |  |  | It can be found in light woodland of all kinds and as a pioneer species over fallen dead trees or in clearcuttings, and at the edge of forests or at the sides of roads. | |
|  |  |  |  |  |  |  | |
| * Grove of trees not separated into core and edge within the habitat observation, thus edge of grove of trees added here. | | | | | | |  |
| High certainty that plants are important and can be localised into one habitat | | | | | | |  |
| High certainty that plants are important, localisation is interplay of hive location, pollen availability in pollen pellets and honey | | | | | | |  |
| Discrepancy between importance from habitat observation and honey and pollen analysis | | | | | | |  |
| Should be included due to knowledge on bee behaviour in foraging. Detailed description in BEESCOUT manual. | | | | | | |  |
| According to literature suggestions | | | | | | |  |
| **Arable crop** |  |  |  |  |  |  |  |
| Only found in treatment site and this only in early assessment of pollen pellets | | | | | | |  |

As mentioned in the main manuscript, a detailed habitat analysis and pollen pellet and honey analysis was not carried out during the experiment after hives were relocated to the second landscape. Thus the characterisation of the landscape in terms of what plant to place in the habitat type is reliable on expert judgement and open literature. We kept OSR as potential food source as it was shown to have been important in landscape L1. *Trifolium repens* was used as food source in landscape L2 because this plant was previously used in BEEHAVE and was shown to be important for bees and is abundantly available in grassland (that can be found frequently in gardens and road sides) (2, 3). *Taraxacum agg.* was used because it is also one of the largest nectar provider for bees (2, 3) and was found in the pollen and honey analysis from samples taken when the hives were located in Landscape 1. The literature suggests that more than 80% of pollen found on bees collected in autumn was ivy (12).

**Additional information on varroa treatment during the field study**

At the beginning of the experiment the infestation of honey bee colonies by *Varroa* mites was relatively low with rates of 0.00 – 0.47 fallen mites per day and then increased exponentially over time. The *Varroa* treatments in hives with flumethrin (experimental day 100 and 160), was not successful because of flumethrin resistant mites within all colonies of all study locations. Since the treatment with flumethrin did not decrease the rate of infestation with the varroa mites, resistance tests were conducted. Furthermore, the efficiency of flumethrin was tested against CheckMite® strips *Varroa* resistance was conducted under non-GLP conditions. A contact test and a test of altered channel proteins which cause flumethrin resistance of varroa mites was conducted to demonstrate the resistance of the mites in the hives. The contact test conducted after both flumethrin treatments on mites from the colonies revealed that only 10 and 1.3% of the mites were affected by the treatment. The test of altered channel proteins revealed that 69 out of 71 mites tested (97.2%) carried the resistance allele either homozygous or heterozygous. Therefore, we assumed for the modelling exercise that no varroa treatment was conducted as it did not show any significant impact on the bee colonies.


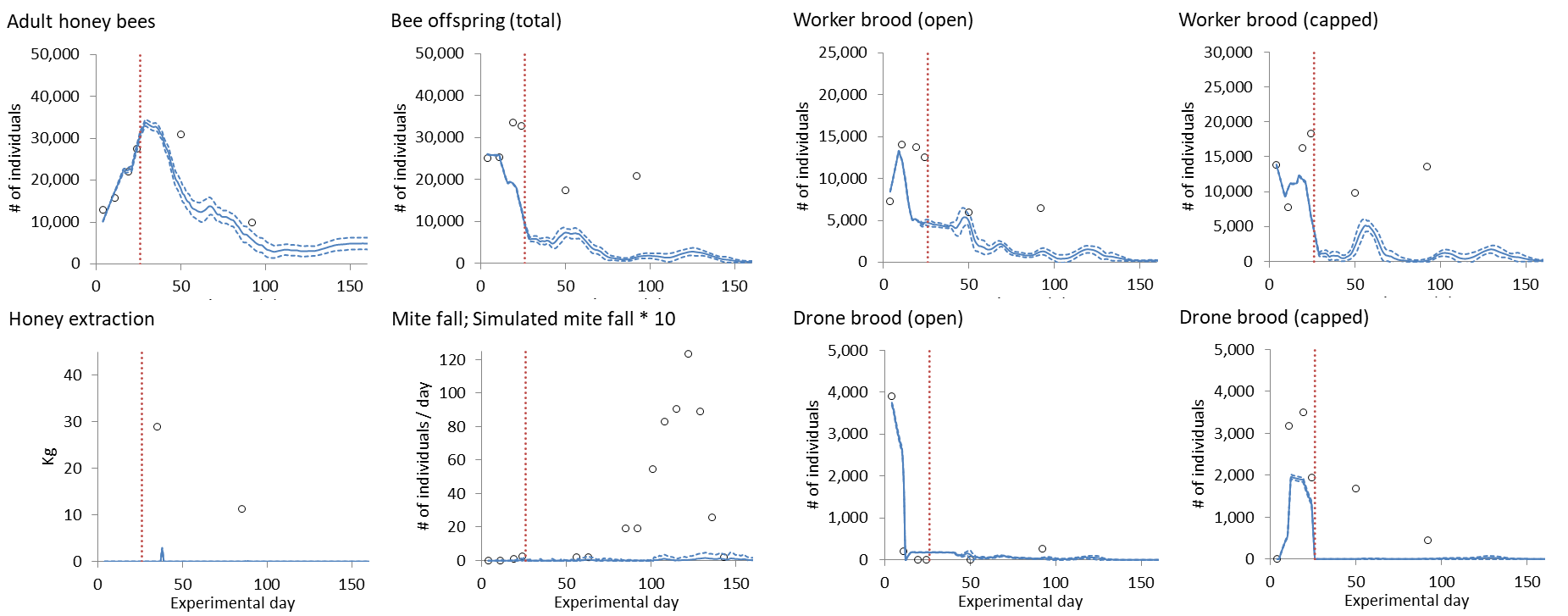


Figure S1: Measured (data points (N=1)) and Simulated (blue lines: average and 95% tolerance interval (N=200)) population dynamics, honey production and mite fall for the colony CA-1. The red dashed line indicates the point where hives were relocated.


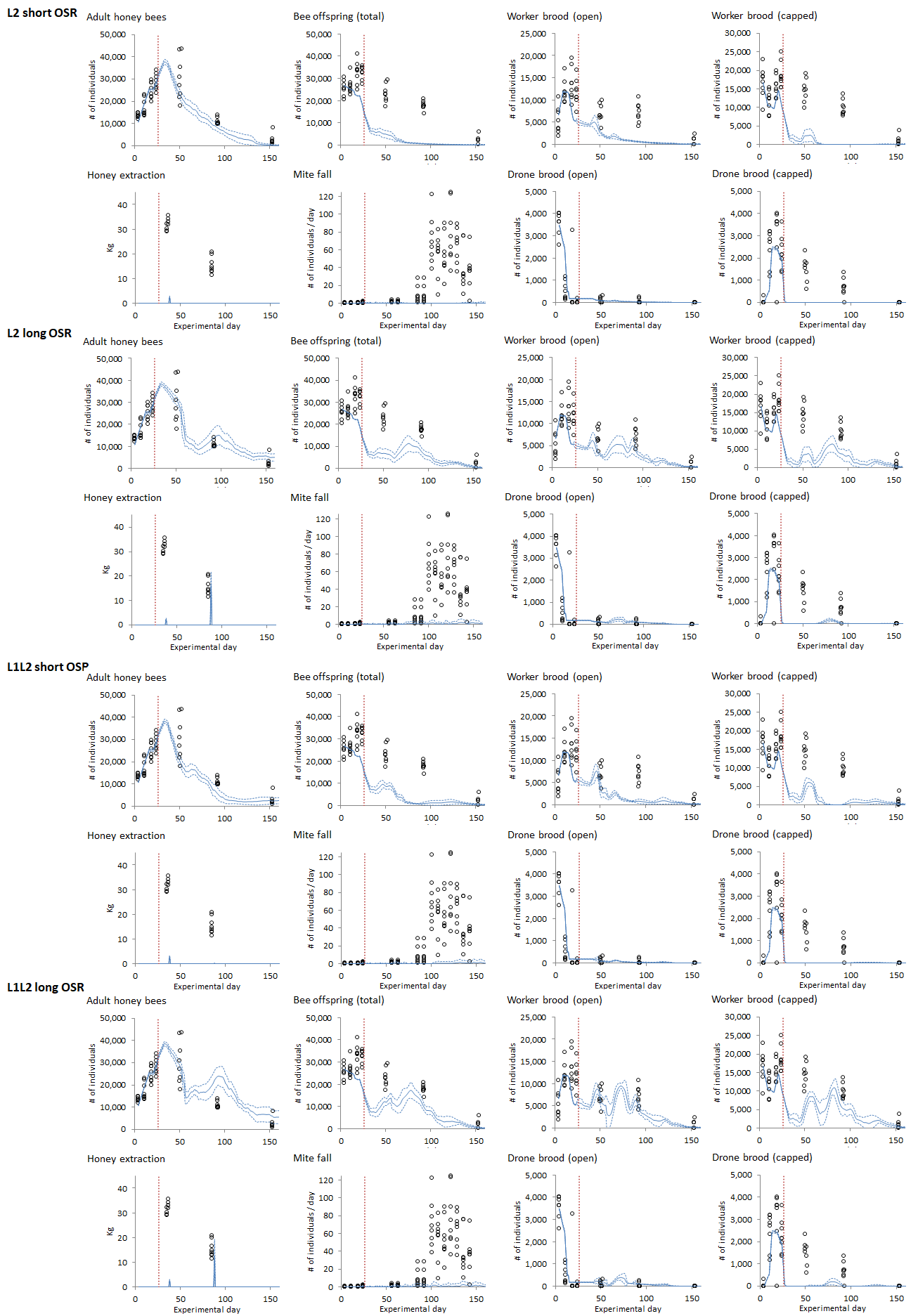


Figure S2: Measured (data points (N=8)) and Simulated (blue lines: average and 95% tolerance interval (N=200)) population dynamics, honey production and mite fall for four different variations of the landscape characterisation. The red dashed line indicates the point where hives were relocated.


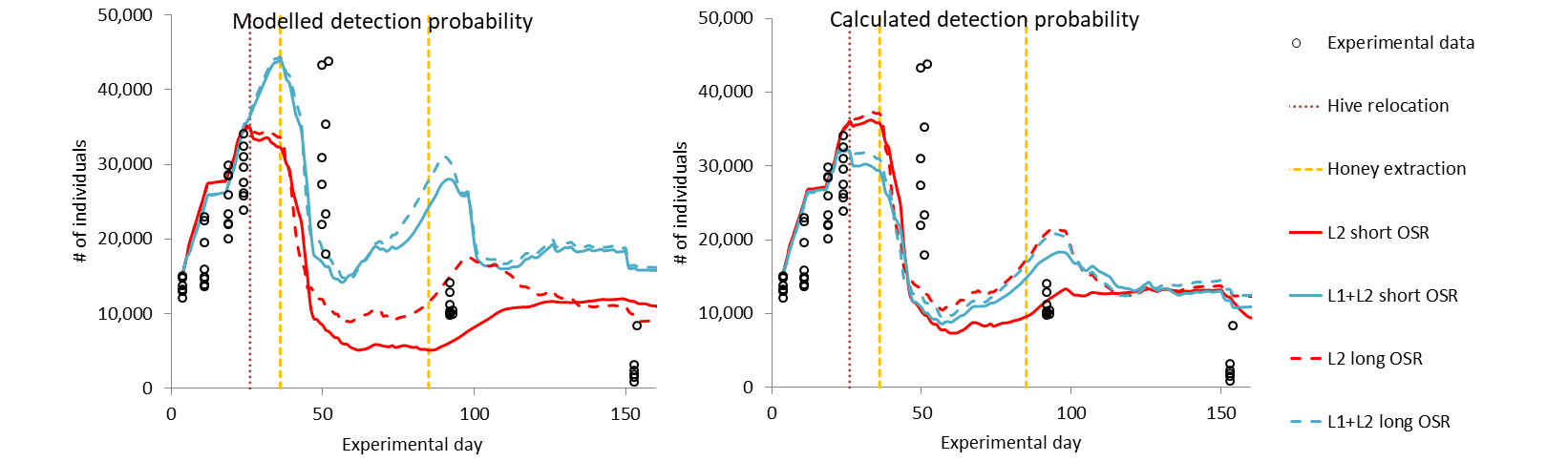


**Figure S3**: Measured and simulated number of adult bees over time. Experimental data illustrate the individual replicate colonies. Simulations for the different landscape characterisation strategies with *Tarraxacum* *agg*. as most melliferous food source are illustrated as average (N=200). Simulations were conducted using the average colony starting conditions, the “random location” scouting strategy and either the modelled habitat detection probability (left) or the calculated detection probability (right). Movement of hives between the landscapes L1 and L2 is indicated by the red dotted line. The orange dotted lines indicate the time where honey was extracted from the hives.


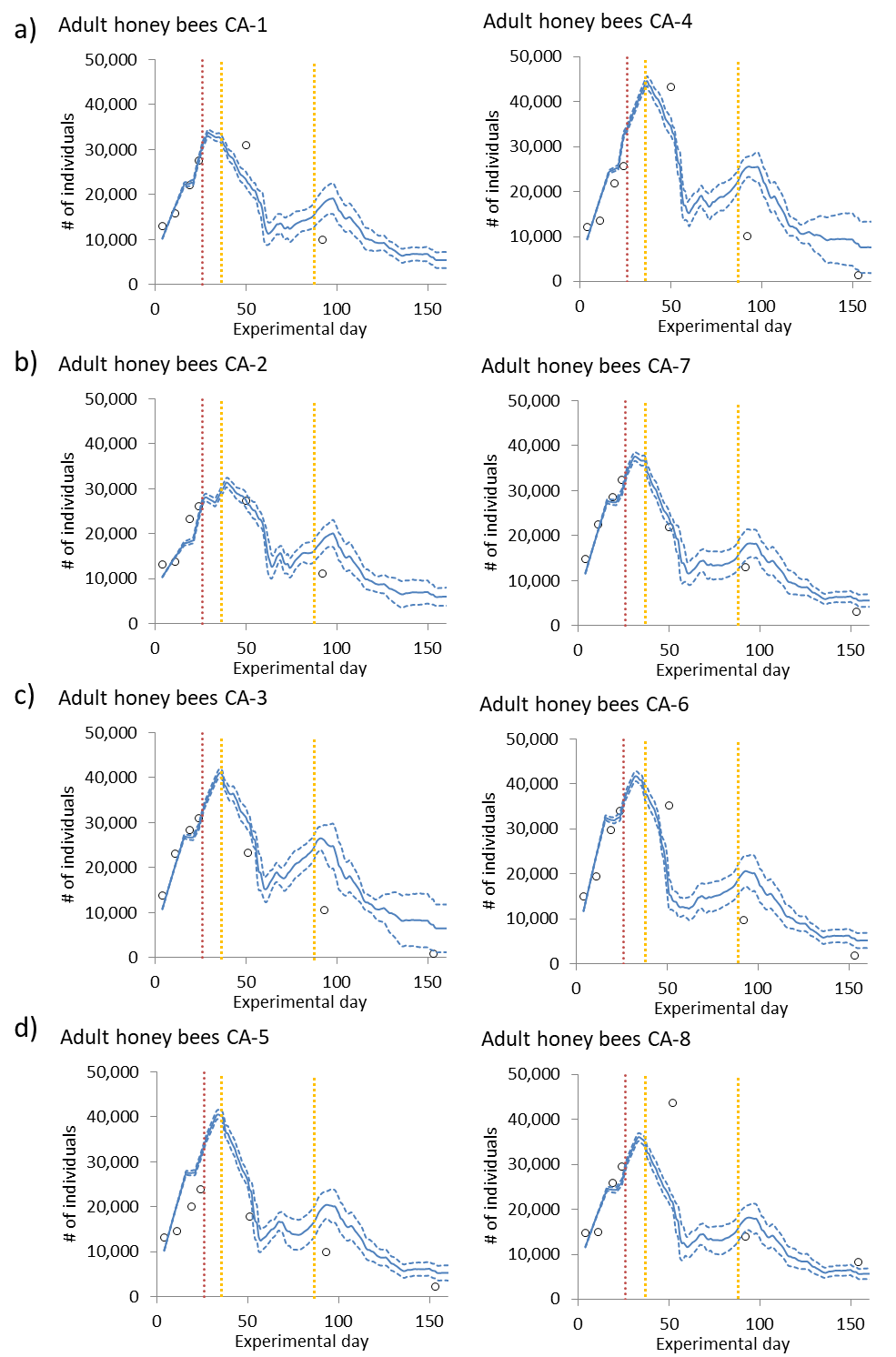


Figure S4: Measured and simulated number of adult bees over time using the long OSR flowering period. Experimental data illustrate the individual hive. Simulations for the landscape characterisation strategy L1+L2 with the hive locations a, b, c, and d within the landscape L2 are illustrated as average with the 95% tolerance interval (N=200). Simulations were conducted using the individual colony starting conditions, the individual hive locations in L1 and L2, and the the “random location” scouting strategy. Movement of hives between the landscapes L1 and L2 is indicated by the red dotted line. The orange dotted line indicates the time where honey was extracted from the hives.


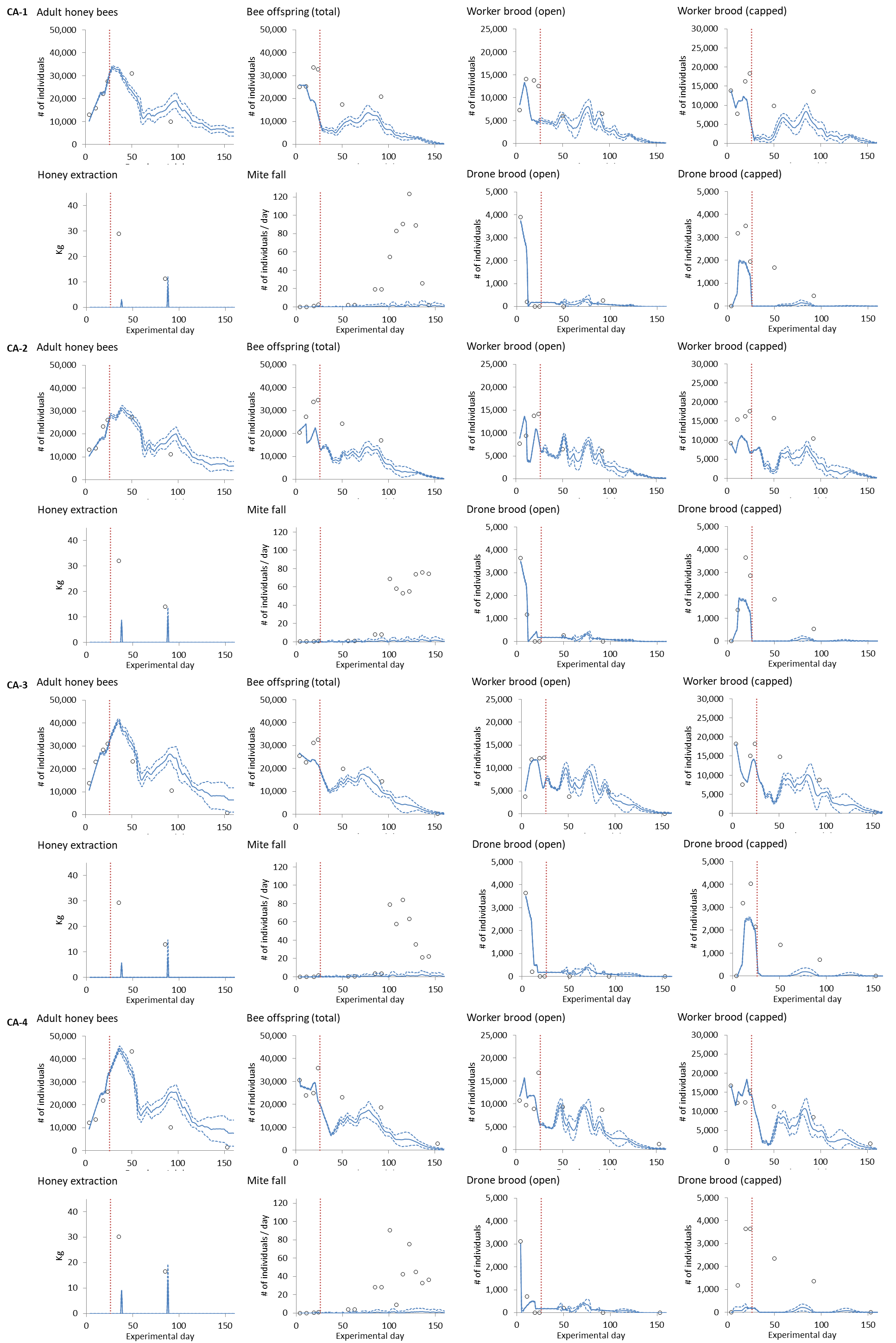


Figure S5 part 1: see the description for Figure S5 continued.


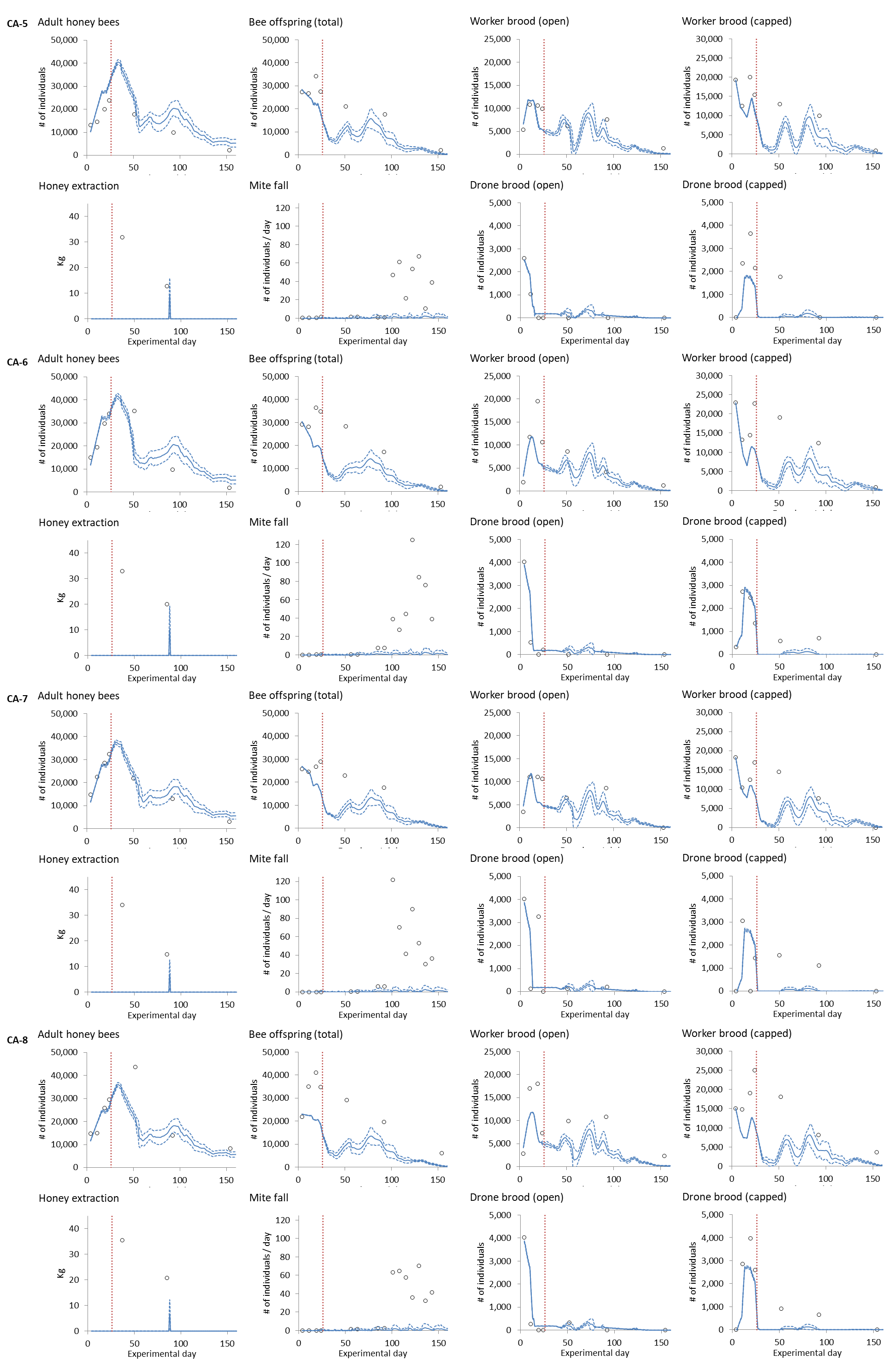


Figure S5 continued: Measured and Simulated (blue lines: average and 95% tolerance interval (N=200)) population dynamics, honey production and mite fall for the 8 different control hives CA-1 to CA-8. The simulations were conducted using the in BEESCOUT according to the integrated “random location” search mode. The red dashed line indicates the point where hives were relocated.

Alterations made to the BEEHAVE code

Added to globals:

DayShifted

exp-added-food

exp_RemainingHoney_kg

Added to Setup just after clear-all:

ifelse exp_input_initial_conditions = true

[set N_INITIAL_BEES round exp_init_IHbees]

[set N_INITIAL_BEES round N_INITIAL_BEES]

Added to Setup just before end:

if exp_input_initial_conditions = true

[InitialConditionsProc

CreateCohortsProc]

Added to DailyUpdateProc:

ifelse exp_input_initial_conditions = false

[ set Day round (ticks mod 365.00001) ]

[set DayShifted round (ticks mod 365.00001) + exp_StartDay

set Day round (DayShifted mod 365.00001) ]

Added to GenericPlottingProc:

if plotChoice = "AGA own" and ticks > 1

[create-temporary-plot-pen "Total adults"

set-plot-pen-color black

plot (TotalForagers + TotalIHbees + TotalDrones)

create-temporary-plot-pen "Total brood"

set-plot-pen-color brown

plot (TotalEggs + TotalLarvae + TotalPupae + TotalDroneEggs + TotalDroneLarvae + TotalDronePupae)

create-temporary-plot-pen " Capped worker brood"

set-plot-pen-color blue

plot (TotalPupae)

create-temporary-plot-pen " Open worker brood"

set-plot-pen-color green

plot (TotalEggs + TotalLarvae)

create-temporary-plot-pen " Capped drone brood"

set-plot-pen-color red

plot (TotalDronePupae)

create-temporary-plot-pen " Open drone brood"

set-plot-pen-color orange

plot (TotalDroneEggs + TotalDroneLarvae)

create-temporary-plot-pen " Total worker brood"

set-plot-pen-color violet

plot (TotalEggs + TotalLarvae + TotalPupae)

create-temporary-plot-pen " Total drone brood"

set-plot-pen-color yellow

plot (TotalDroneEggs + TotalDroneLarvae + TotalDronePupae)

create-temporary-plot-pen "Honey Harvested"

set-plot-pen-color gray

plot (HarvestedHoney_kg)

create-temporary-plot-pen "Mite fall"

set-plot-pen-color lime

plot (DailyMiteFall * 10) ]

Added to BeekeepingProc:

ifelse Day = exp_harvest-day1 or Day = exp_harvest-day2 or Day = exp_harvest-day3 or Day = exp_harvest-day4

and exp_Honey-harvest = true

[

if day = exp_harvest-day1 [set exp_RemainingHoney_kg exp_remaining-honey1]

if day = exp_harvest-day2 [set exp_RemainingHoney_kg exp_remaining-honey2]

if day = exp_harvest-day3 [set exp_RemainingHoney_kg exp_remaining-honey3]

if day = exp_harvest-day4 [set exp_RemainingHoney_kg exp_remaining-honey4]

ifelse (HoneyEnergyStore / (ENERGY_HONEY_per_g * 1000)) - exp_RemainingHoney_kg > 0

[

set HarvestedHoney_kg (HoneyEnergyStore / (ENERGY_HONEY_per_g * 1000)) - exp_RemainingHoney_kg

set HoneyEnergyStore HoneyEnergyStore - (HarvestedHoney_kg * ENERGY_HONEY_per_g * 1000)

]

[set HarvestedHoney_kg 0]

set TotalHoneyHarvested_kg TotalHoneyHarvested_kg + HarvestedHoney_kg

output-type "Honey harvest on day "

output-type ceiling (day mod 30.4374999)

output-type "."

output-type floor(day / (365.25 / 12)) + 1

output-type "."

output-type ceiling (ticks / 365)

output-type ". Amount [kg]: "

output-type precision HarvestedHoney_kg 1

output-type " total honey harvested: "

output-print precision TotalHoneyHarvested_kg 1

ask Signs with [shape = "honeyjar"]

[

show-turtle

set label precision HarvestedHoney_kg 1

]

]

[set HarvestedHoney_kg 0]

if exp_feeding-shedule = true and day = exp_feeding-day1 or day = exp_feeding-day2 or day = exp_feeding-day3 or day = exp_feeding-day4

[

if day = exp_feeding-day1 [set exp-added-food exp_added-fondant1]

if day = exp_feeding-day2 [set exp-added-food exp_added-fondant2]

if day = exp_feeding-day3 [set exp-added-food exp_added-fondant3]

if day = exp_feeding-day4 [set exp-added-food exp_added-fondant4]

set TotalHoneyFed_kg TotalHoneyFed_kg + exp-added-food

set HoneyEnergyStore HoneyEnergyStore + (exp-added-food * ENERGY_HONEY_per_g * 1000)

output-type "Feeding colony on day "

output-type ceiling (day mod 30.4374999) ; day

output-type "."

output-type floor(day / (365.25 / 12)) + 1 ; month

output-type "."

output-type ceiling (ticks / 365) ; year

output-type " Fondant provided [kg]: "

output-type precision exp-added-food 1

output-type " total food added [kg]: "

output-print precision exp-added-food 1

ask Signs with [shape = "ambrosia"] [ show-turtle]

]

Added procedures:

to InitialConditionsProc

set DayShifted round (ticks mod 365.00001) + exp_StartDay

set Day round (DayShifted mod 365.00001)

set honey_store_init exp_init_honey

set pollen_store_init exp_init_pollen

set HoneyEnergyStore (HONEY_STORE_INIT * ENERGY_HONEY_per_g)

set IdealPollenStore_g POLLEN_STORE_INIT

set MAX_HONEY_ENERGY_STORE MAX_HONEY_STORE_kg * ENERGY_HONEY_per_g * 1000

set PollenStore_g POLLEN_STORE_INIT

set NewWorkerEggs exp_init_eggs

set NewDroneEggs exp_init_eggs_drone

set NewWorkerLarvae exp_init_larvae

set NewDroneLarvae exp_init_larvae_drone

set NewWorkerPupae exp_init_pupae

set NewDronePupae exp_init_pupae_drone

set NewIHbees exp_init_IHbees

set NewForagerSquadronsHealthy (NewIHbees / SQUADRON_SIZE)

set TotalForagers NewForagerSquadronsHealthy * SQUADRON_SIZE

set MORTALITY_DRONE_EGGS 0

set MORTALITY_DRONE_LARVAE 0

set MORTALITY_DRONE_PUPAE 0

set MORTALITY_DRONES 0

set MORTALITY_EGGS 0

set MORTALITY_LARVAE 0

set MORTALITY_PUPAE 0

set MORTALITY_FOR_PER_SEC 0

set MORTALITY_INHIVE 0

end

to CreateCohortsProc

create-eggCohorts 1

[ set number round (exp_init_IHbees )

set age 5

set shape "circle"

setxy 3 0

set color blue

set ploidy 2

;____________________________________________

let x -12

repeat 12

[create-eggCohorts 1

[ set number round (NewWorkerPupae / 12)

set age x

set shape "circle"

setxy 3 0

set color blue

set ploidy 2 ]

set x x + 1]

set x -10

repeat 10

[

create-DroneeggCohorts 1

[

set number round (NewDronePupae / 10)

set age x

set shape "circle"

setxy 3 0

set color blue

set ploidy 2

]

set x x + 1

]

;______________________________________________

set x -17

repeat 5

[

create-eggCohorts 1 ;

[

set number round (NewWorkerLarvae / 5)

set age x

set shape "circle"

setxy 3 0

set color blue

set ploidy 2

]

create-DroneeggCohorts 1 ;

[

set number round (NewDroneLarvae / 5)

set age x

set shape "circle"

setxy 3 0

set color blue

set ploidy 2

]

set x x + 1

]

;_______________________________________

set x -20

repeat 3

[

create-eggCohorts 1 ;

[

set number round (NewWorkerEggs / 3)

set age x

set shape "circle"

setxy 3 0

set color blue

set ploidy 2

]

create-DroneeggCohorts 1 ;

[

set number round (NewDroneEggs / 3)

set age x

set shape "circle"

setxy 3 0

set color blue

set ploidy 2

]

set x x + 1

]

set NewWorkerEggs 0

set NewDroneEggs 0

set NewWorkerLarvae 0

set NewDroneLarvae 0

set NewWorkerPupae 0

set NewDronePupae 0

set NewForagerSquadronsHealthy 0

set x 20

repeat 20

[

if x = 19 [set NewIHbees 0

]

WorkerEggsDevProc

DroneEggsDevProc

NewEggsProc

WorkerLarvaeDevProc

DroneLarvaeDevProc

NewWorkerLarvaeProc

NewDroneLarvaeProc

WorkerPupaeDevProc

DronePupaeDevProc

NewWorkerPupaeProc

NewDronePupaeProc

WorkerIHbeesDevProc

DronesDevProc

NewIHbeesProc

NewDronesProc

MiteProc

CountingProc

set x x - 1

]

set MORTALITY_DRONE_EGGS 0.064

set MORTALITY_DRONE_LARVAE 0.044

set MORTALITY_DRONE_PUPAE 0.005

set MORTALITY_DRONES 0.05

set MORTALITY_EGGS 0.03

set MORTALITY_LARVAE 0.01

set MORTALITY_PUPAE 0.001

set MORTALITY_FOR_PER_SEC 0.00001

set MORTALITY_INHIVE 0.004

end

**References**

1. Becher MA*, et al.* (2016) BEESCOUT: A model of bee scouting behaviour and a software tool for characterizing nectar/pollen landscapes for BEEHAVE. *Ecological Modelling* 340:126-133.

2. Hicks DM*, et al.* (2016) Food for Pollinators: Quantifying the Nectar and Pollen Resources of Urban Flower Meadows. *PLoS ONE* 11(6):e0158117.

3. Baude M*, et al.* (2016) Historical nectar assessment reveals the fall and rise of Britain in bloom. *Nature* 530(7588):85-88.

4. Dötterl S, Glück U, Jürgens A, Woodring J, & Aas G (2014) Floral Reward, Advertisement and Attractiveness to Honey Bees in Dioecious Salix caprea. *PLOS ONE* 9(3):e93421.

5. Gyan KY & Woodell SRJ (1987) Nectar Production, Sugar Content, Amino Acids and Potassium in Prunus spinosa L., Crataegus monogyna Jacq. and Rubus fruticosus L. at Wytham, Oxfordshire. *Functional Ecology* 1(3):251-259.

6. Somme L*, et al.* (2016) Food in a row: urban trees offer valuable floral resources to pollinating insects. *Urban Ecosystems* 19(3):1149-1161.

7. Molina RT, Rodríguez AM, Palaciso IS, & López FG (1996) Pollen production in anemophilous trees. *Grana* 35(1):38-46.

8. Müller A*, et al.* (2006) Quantitative pollen requirements of solitary bees: Implications for bee conservation and the evolution of bee–flower relationships. *Biological Conservation* 130(4):604-615.

9. Dicks LV*, et al.* (2015) How much flower‐rich habitat is enough for wild pollinators? Answering a key policy question with incomplete knowledge. *Ecological Entomology* 40(Insects and Ecosystem Services 28th Symposium of the Royal Entomological Society of LondonS1):22-35.

10. Percival MS (1955) The presentation of pollen in certain angio-sperms and its collection by Apis mellifera. *New Phytologist* 54(3):353-368.

11. BUTLER CG (1945) The Influence of Various Physical and Biological Factors of the Environment on Honeybee Activity. An Examination of the Relationship between Activity and Nectar Concentration and Abundance. *Journal of Experimental Biology* 21(1-2):5-12.

12. Garbuzov M & Ratnieks FLW (2014) Ivy: an underappreciated key resource to flower-visiting insects in autumn. *Insect Conservation and Diversity* 7(1):91-102.

13. Couvillon MJ*, et al.* (2015) Busy Bees: Variation in Insect Flower-Visiting Rates across Multiple Plant Species. *Psyche* 2015:7.

14. Schmickl T & Crailsheim K (2007) HoPoMo: A model of honeybee intracolonial population dynamics and resource management. *Ecological Modelling* 204(1):219-245.
